# Case Report: Neuroblastoma-Like Schwannoma in a Domestic Short-Haired Cat

**DOI:** 10.3389/fvets.2022.905302

**Published:** 2022-06-17

**Authors:** Vivian S. Chen, Andrew W. Bollen, Paola Marco-Salazar, Robert J. Higgins, Sílvia Sisó

**Affiliations:** ^1^Charles River Laboratories, Inc., Durham, NC, United States; ^2^Neuropathology Division, Department of Pathology, School of Medicine, University of California, San Francisco, San Francisco, CA, United States; ^3^Department of Medicine and Animal Surgery, School of Veterinary Medicine, Autonomous University of Barcelona (UAB), Bellaterra, Spain; ^4^Department of Pathology, Microbiology and Immunology, School of Veterinary Medicine, University of California, Davis, Davis, CA, United States; ^5^Translational Imaging and Pathology, Codiak BioSciences, Cambridge, MA, United States

**Keywords:** schwannoma, neurilemoma, peripheral nerve sheath tumor, neuroblastoma, cat

## Abstract

An axillary mass was detected in a 6-year-old, neutered, male, domestic short-haired cat during a wellness exam. Gross examination following surgical removal revealed a discrete, deep subcutaneous, discoid mass that was between 0.5- and 0.7-cm-in-diameter and diffusely firm and white. Histologically, the mass was well-demarcated, partially encapsulated, and expanded the panniculus carnosus. It was composed of tightly packed, giant rosettes of radially arranged fusiform cells stacked in one to 10 layers with peripherally palisading nuclei and with centrally oriented, fibrillary, cytoplasmic processes, and collagenous fibers. Laminin immunoreactivity and ultrastructural examination highlighted a continuous basal lamina outside the plasma membrane of each neoplastic cell. Neoplastic cells were immunoreactive for GFAP, S100, periaxin, and Sox-10 and were immunonegative for synaptophysin, smooth muscle actin, and pancytokeratin. Collective findings were consistent with a diagnosis of neuroblastoma-like schwannoma. This is the first veterinary report of this rare variant of benign schwannoma.

## Introduction

In cats, cutaneous schwannomas, which are grouped within the entity of benign peripheral nerve sheath tumors (PNST), occur predominantly in the head or neck and, with less frequency, in the limbs or the trunk (1). Recent studies of a large feline case series found that PNST comprised at least 7% of all skin tumors and are more common than previously reported (2). Schwannomas are usually characterized histologically by fusiform cells organized in a storiform pattern with nuclear palisading, and with alternating patterns of compact, cellular (Antoni type A) to loosely arranged, myxoid (Antoni type B) streams and fascicles of spindloid cells (3). However, classification of schwannomas in domestic animal species is challenging due to the small number of histological studies, often the absence of distinct PNST features, and the lack of a diagnostic panel of spatial biomarkers for confirming the histogenesis of neoplastic cells (3). Moreover, morphologic variants of schwannomas that are recognized in human medicine, such as “ancient” schwannoma or epithelioid schwannoma, have been rarely reported in domestic animals (3, 4). In the cat, schwannomas have been lumped broadly under the category of benign or malignant PNST without any distinction between schwannomas, perineuriomas, or neurofibromas (1, 2). Neuroblastoma-like schwannoma is a variant of benign schwannoma that has rarely been described in people and not previously in a domestic animal species (4). This variant has unique histomorphologic features, and the immunophenotypic characteristics are consistent with a histogenesis from Schwann cells.

## Case Presentation

A 6-year-old, neutered, male, domestic short-haired cat was presented to the William R. Pritchard Veterinary Medical Teaching Hospital (VMTH), University of California – Davis, California, USA, for a wellness examination, including an evaluation of an axillary mass that was first noted by the owners 2 weeks prior to the visit. Prior medical history included feline herpes virus-1 infection and symblepharon of the left eye as a kitten, and mild overconditioning and mild dental disease as an adult.

The mass was palpated as approximately 1-cm-in-diameter, round, firm and partially adhered to the muscle and connective tissue of the left lateral thoracic wall and axilla. Examination of a fine needle aspirate of the mass was tentatively diagnosed as a feline basal cell tumor and the owners elected surgical removal of the mass. It was removed ~1 week later with 1- to 2-cm lateral margins and with one fascial plane deep to the mass. At surgery, the mass was discoid, between 0.5- and 0.7-cm-in-diameter and diffusely firm and white. The fresh specimen was trimmed, and most sections were immediately immersion-fixed in 10% buffered formalin for 24 h and then processed and embedded in paraffin wax. Additional tissue sections were submitted in Karnovsky's fixative to California Animal Health and Food Safety Services Laboratory Systems (CAHFS, Davis, CA) and routinely processed for transmission electron microscopic examination. For light microscopy, 5-μm tissue sections were stained with haematoxylin and eosin (H&E), Masson's trichrome stain or processed for immunohistochemistry (IHC) using 1-h incubation periods with one of several primary antibodies (Table 1). The panel of primary antibodies included those to laminin, glial fibrillary acidic protein (GFAP), S-100, periaxin, Sox-10, smooth muscle actin (SMA), synaptophysin and Ki-67. Table 1 summarizes details of the vendors, the different IHC reagents and antigen unmasking protocols used for each antibody. Negative controls in which either diluent or normal serum was substituted for the respective primary antibody were included in the study, and standardized positive control tissue, as used in the UCD VMTH histology laboratory, was included for each antibody where appropriate.

**Table 1:** Panel of antigenic markers used by immunohistochemistry.

**Primary antibody**	**Provider; Location (Catalog number)**	**Pre-treatment**	**Primary antibody dilution**	**Secondary antibody**	**Detection system (catalog number)**
Synaptophysin (SY38, SYN)	Dako; Carpinteria, CA, USA (M0776)	Heat induced epitope retrieval (HIER) Steamer-30'	1:80	Mouse Dako Envision+ system-HRP (K4006)	Vector NovaRed (SK-4800)
Glial fibrillary acidic protein (GFAP)	Dako; Carpinteria, CA, USA (Z0334)	Proteinase K-5'	1:600	Rabbit Dako Envision+ system-HRP (K4006)	Vector NovaRed (SK-4800)
Laminin (LAM)	BioGenex, (PU078-UP)	0.2% protease, 37C, 10'	1:40	Rabbit Biocare Medical 4+ Detection System; Streptavidin-HRP	Vector NovaRed (SK-4800)
Anti-human Periaxin	Sigma-Aldrich (57716)	(HIER) Steamer-30'	1:1000	Rabbit Biocare Medical 4+ Detection System; Streptavidin-HRP	Vector NovaRed (SK-4800)
Anti-human Sox-10	Santa Cruz (SC-17342)	(HIER) Steamer-30'	1:100	Rabbit Biocare Medical 4+ Detection System; Streptavidin-HRP	Vector NovaRed (SK-4800)
S-100	Vector VP-S276	(HIER) Steamer-30'	1:600	Rabbit Biocare Medical 4+ Detection System; Streptavidin-HRP	Vector NovaRed (SK-4800)
Smooth Muscle Actin (1A4; SMA)	BioGenex (MU128-UC)	Steam EDTA-9, 30'	1:300	Mouse Dako Envision+ system-HRP (K4006)	Vector NovaRed (SK-4800)
Ki-67	Dako; Carpinteria, CA, USA (M7240)	(HIER) Steamer-30'	1:40	Mouse Biocare Medical 4+ Detection System; Streptavidin-HRP	Vector NovaRed (SK-4800)

**Figure 1:**
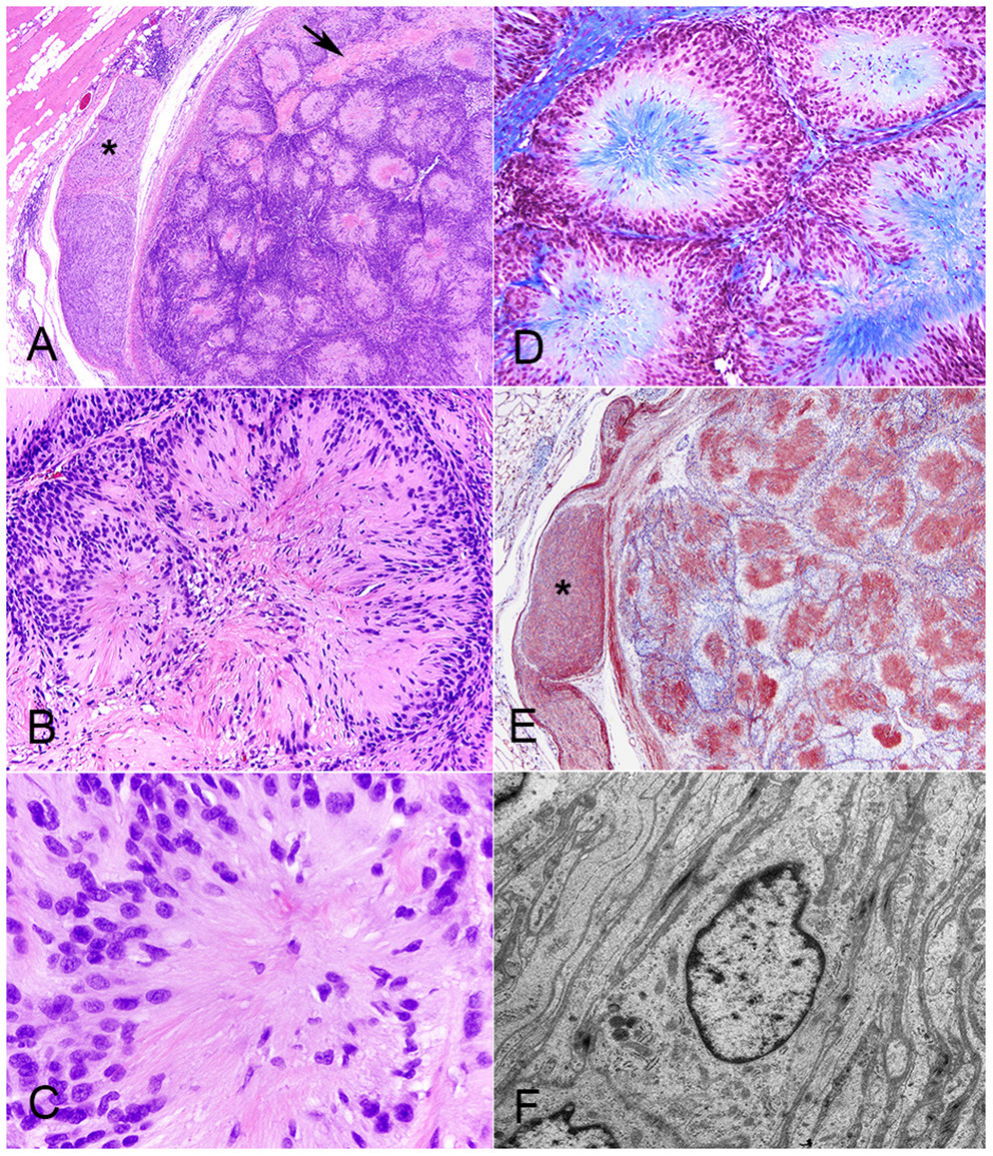
Neuroblastoma-like schwannoma, cat, subcutaneous mass. The mass is composed of multiple lobules that are separated by variably thick bands of dense connective tissue (arrow). Note the hypercellular peripheral nerve fascicles (asterisk) in the vicinity of the mass, which is partially encapsulated by the epineurium. H&E stain **(A)**. The lobules are composed of multiple, tightly packed, 5–300-μm-in-diameter, fibrillar, giant rosettes that exhibited a characteristic cellular polarity with a peripheralized nucleus and centrally oriented cytoplasm. Note that there is nuclear palisading. H&E stain **(B)**. Each individual giant rosette is characterized by radially-arranged fusiform cells with round to oval, lymphocyte-like nuclei and eosinophilic fibrillary cytoplasm. H&E **(C)**. Giant rosettes contain a centralized collagen core (in blue). Masson's trichrome stain **(D)**. Each fibrillar and eosinophilic cytoplasmic neoplastic process, similar to each Schwann cell in healthy peripheral nerves (asterisk), diffusely and strongly immunoreacts to laminin. Immunohistochemistry for laminin **(E)**. Each neoplastic cell is coated by a well-formed, continuous, 50 nm thick, electron-dense basal lamina investing long and interdigitating cytoplasmic processes. The cytoplasm contains a flattened, often invaginated nucleus, microfibrils and sparse organelles with a few mitochondria, ribosomes, and lysosome-like granules. TEM micrograph **(F)**.

Histologically, there was a discrete, multilobular and partially encapsulated mass that focally expanded the panniculus carnosus in the vicinity of hypercellular peripheral nerve fascicles. The stretched capsule surrounding the mass consisted of epineurium and a few residual nerve fibers (Figure 1A). The lobules, which were separated by variably thick bands of dense connective tissue, varied in size, and were composed of multiple tightly packed, 5- to 300-μm-in-diameter, fibrillar, giant rosettes. Each rosette was characterized by radially-arranged, fusiform cells with indistinct cell borders, and twisted nuclei stacked in one to 10 layers at the periphery with often prominent nuclear palisading (Figures 1B,C). The center of the rosettes was composed of various amounts of trichrome-positive collagen fibrils (Figure 1D) and of indistinct fibrillar and eosinophilic cytoplasmic processes, which were laminin immunoreactive (Figure 1E). The nuclei were round to oval and occasionally indented with mostly stippled chromatin and variably distinct nucleoli. Anisocytosis and anisokaryosis were mild, and mitotic figures were occasional (3 mitotic figures in 10 400x fields). Variably areas of intratumoral coagulative necrosis were occasional throughout. Very small numbers of lymphocytes, plasma cells, and rare neutrophils were scattered within the mass, mostly surrounding lobules. Ultrastructural examination of neoplastic cells corroborated a well-formed, continuous basal lamina investing long and interdigitating cytoplasmic processes (Figure 1F). Multifocally, variable amounts of collagen fibrils were deposited extracellularly (Supplementary Figure 1). Neoplastic cells exhibited sparse organelles with a few mitochondria, ribosomes, and lysosome-like granules and had twisted and indented nuclei and variable numbers of nucleoli. Re-examination of the initial cytological smear (Supplementary Figure 1) confirmed the pattern of giant rosettes with piling of elongated nuclei at the periphery and centrally-oriented, fibrillary processes.

**Figure 2:**
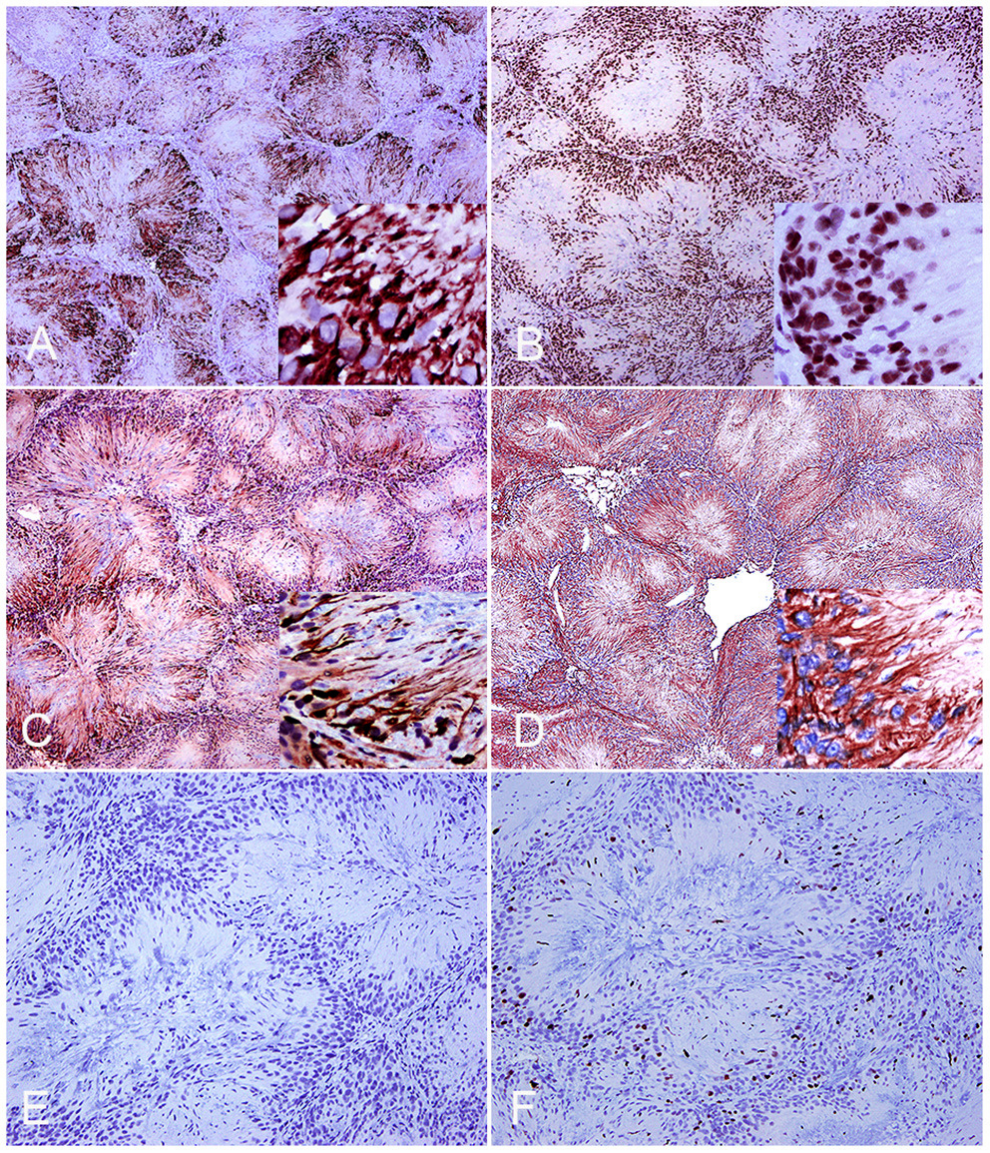
Neuroblastoma-like schwannoma, cat, subcutaneous mass. Immunohistochemistry. Neoplastic Schwann cells cytoplasmic processes diffusely and strongly immunoreact to periaxin **(A)**. One hundred percent of nuclei of neoplastic cells are strongly positive for Sox-10 (inset) **(B)**. Neoplastic Schwann cell cytoplasmic processes immunoreact to S-100 **(C)**. Neoplastic fibrillary cytoplasmic processes are strongly labeled with glial fibrillary acid protein **(D)**. Immunohistochemistry for synaptophysin confirms lack of immunoreactivity of neoplastic cells **(E)**. Approximately 10% of the cells display nuclear ki-67 (MIB-1) immunostaining **(F)**.

A final diagnosis of neuroblastoma-like schwannoma was made based on the cytomorphological features and the strong immunoreactivity to laminin, which characteristically highlighted basal lamina around each neoplastic Schwann cell. Additional immune markers revealed that neoplastic cells were also strongly immunoreactive to GFAP, S-100, periaxin, and Sox-10 but with no immunoreactivity to synaptophysin and SMA (Figures 2A–F). The proliferative index was 10, as calculated by the proportion of Ki-67-positive nuclei in a total count of 2,000 cells. Previous studies on feline benign PNSTs reported neoplastic cells as mostly consistently immunoreactive for laminin, variably immunoreactive to S-100 and GFAP, and minimally to SMA (1, 2). In this study, two additional relatively novel Schwann cell markers, periaxin and Sox-10, supported the histogenesis of this neoplasm as a schwannoma. Both markers have been evaluated in canine PNSTS together with laminin (3). Periaxin is required for maintenance of the myelin sheath that is formed from the cell membrane of Schwann cells (5), and Sox-10 is expressed in some cells of the Schwann cell lineage of peripheral nerves and enhances Schwann cell responsiveness to axonal neuregulin and cell survival (6).

Rosette formation with strong immunolabeling of neoplastic cells with neuronal-specific immune biomarkers such as synaptophysin, is a characteristic feature of neuroblastomas (3). This lack of immunoreactivity, together with the other results of immunophenotyping, confirmed a neuroblastoma-like schwannoma as the definitive diagnosis. Consistent with reports in people, this rare variant of schwannoma has strong S-100 immunoreactivity and primarily occurs as a solitary and sporadic mass in the dermis and subcutaneous tissues with scattered reports in other sites, such as the orbit and as a pleural mass in the posterior mediastinum (7–10). A total of 25 neuroblastoma-like schwannomas have been reported in people, preferentially female and aged from 16 to 64 years old (8, 10, 11). Differential diagnoses include the hyalinizing spindle cell tumor with giant rosettes, a variant of low-grade fibromyxoid sarcoma, which despite mimicking the histomorphology of neuroblastoma-like schwannoma, has a malignant behavior and is not immunoreactive to S-100 (10).

In summary, this is the first report of neuroblastoma-like schwannoma in a domestic animal. The histologic hallmark of this variant of schwannomas is the uniform rosette histoarchitecture. Despite they can resemble Homer-Wright rosettes, they are much larger than those seen in neuroblastomas, lack immunoreactivity to synaptophysin and display strong immunoreactivity to Schwann cell markers like laminin, S-100 and Sox-10. Also, their centers appeared to be composed of collagen rather than neurites, as demonstrated by histochemical stains and immunohistochemistry. Histological features that favored a benign diagnosis in this case include encapsulation, lack of mitotic figures and absence of necrosis. At a 3-month follow-up examination, a computerized tomography (CT) scan demonstrated mild contrast enhancement of the left ventrolateral thoracic body wall subcutaneous tissue deep to the previous surgical scar, suggestive of either granulation tissue or recurrent neoplasia. However, further contact for follow-up with the cat has been lost.

## Data Availability Statement

The raw data supporting the conclusions of this article will be made available by the authors, without undue reservation.

## Author Contributions

VC was the UC Davis anatomic-pathology resident who received and work-out the case. PM-S was a visiting Ph.D. student who work-out the IHC of the case. SS was the UC Davis Neuropathology Fellow who act as the reviewer neuropathologist and lead the diagnostic immunohistochemical approach. RH and AB were the expert opinion senior neuropathologists who help diagnose the case. All authors contributed to the diagnosis of the case and concept of the case report and writing.

## Funding

This study was initiated and completed at the VMTH of UC Davis during VC's last year of residency in anatomic pathology and SS as a Fellow in Neuropathology, UC Davis, Davis. This study was funded by the Center for Companion Animal Health at UC Davis (CCAH Alloc.# 2013-35-R siso). PM-S was supported by an AGAUR Ph.D. visiting grant [2012 BE100584] from the Generalitat de Catalunya, Barcelona, Catalunya, Spain.

## Conflict of Interest

VC was employed by Charles River Laboratories, Inc. The remaining authors declare that the research was conducted in the absence of any commercial or financial relationships that could be construed as a potential conflict of interest.

## Publisher's Note

All claims expressed in this article are solely those of the authors and do not necessarily represent those of their affiliated organizations, or those of the publisher, the editors and the reviewers. Any product that may be evaluated in this article, or claim that may be made by its manufacturer, is not guaranteed or endorsed by the publisher.

## Supplementary Material

Supplementary Figure 1 Neuroblastoma-like schwannoma, cat, subcutaneous mass. **(A)** Multifocally, variable amounts of scaffolds of ~53 nm thick collagen fibrils (inset) form stacks along processes and in between basal laminae. Transmission electron microscopy. **(B)** A cytological smear of the mass recapitulates the giant rosettes with prominent cellular polarity. Note the piling of oval nuclei at the periphery with the centrally oriented fibrillary processes. Wright Giemsa stain.
