# Supplementary figures and images for: Case Report: Neuroblastoma-Like Schwannoma in a Domestic Short-Haired Cat
Source: Front Vet Sci. 2022 Jun 17;9:905302. doi: 10.3389/fvets.2022.905302 (PMC9247645; doi:10.3389/fvets.2022.905302)

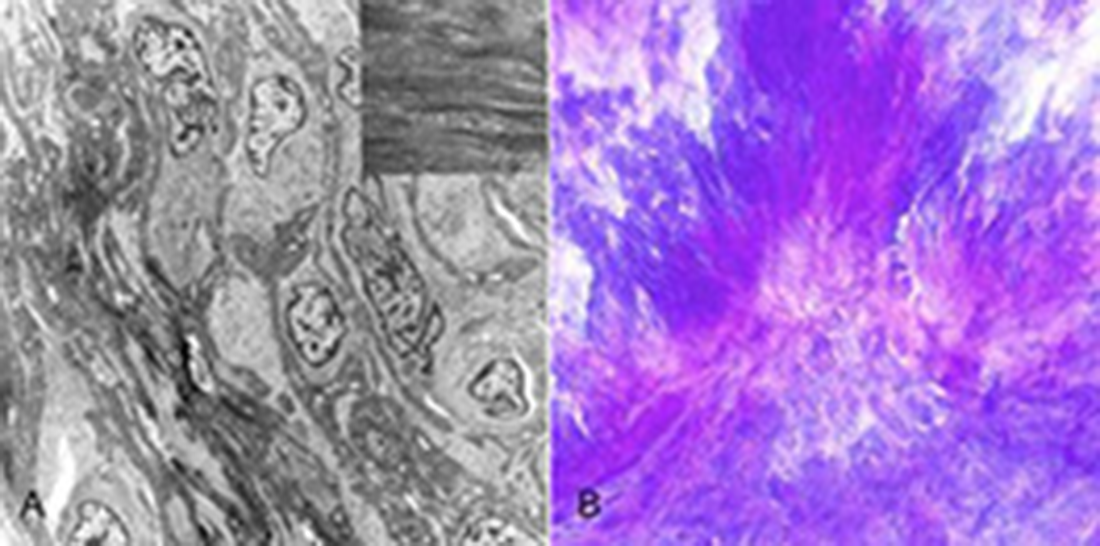

Supplement: Supplementary Figure 1 — Neuroblastoma-like schwannoma, cat, subcutaneous mass. (A) Multifocally, variable amounts of scaffolds of ~53 nm thick collagen fibrils (inset) form stacks along processes and in between basal laminae. Transmission electron microscopy. (B) A cytological smear of the mass recapitulates the giant rosettes with prominent cellular polarity. Note the piling of oval nuclei at the periphery with the centrally oriented fibrillary processes. Wright Giemsa stain. [file Image_1.TIF]
